# The potential utility of fecal (or intestinal) microbiota transplantation in controlling infectious diseases

**DOI:** 10.1080/19490976.2022.2038856

**Published:** 2022-03-01

**Authors:** Rohma Ghani, Benjamin H. Mullish, Lauren A. Roberts, Frances J. Davies, Julian R. Marchesi

**Affiliations:** a Division of Digestive Diseases, Department of Metabolism, Digestion and Reproduction, Imperial College London, London, UK; b MRC Centre for Molecular Bacteriology and Infection, Imperial College London, London, UK

**Keywords:** Intestinal microbiota transplantation, fecal microbiota transplantation, intestinal microbiota, gut microbiota, infectious diseases, antibiotic resistance, IMT, FMT

## Abstract

The intestinal microbiota is recognized to play a role in the defense against infection, but conversely also acts as a reservoir for potentially pathogenic organisms. Disruption to the microbiome can increase the risk of invasive infection from these organisms; therefore, strategies to restore the composition of the gut microbiota are a potential strategy of key interest to mitigate this risk. Fecal (or Intestinal) Microbiota Transplantation (FMT/IMT), is the administration of minimally manipulated screened healthy donor stool to an affected recipient, and remains the major ‘whole microbiome’ therapeutic approach at present. Driven by the marked success of using FMT in the treatment of recurrent *Clostridioides difficile* infection, the potential use of FMT in treating other infectious diseases is an area of active research. In this review, we discuss key examples of this treatment based on recent findings relating to the interplay between microbiota and infection, and potential further exploitations of FMT/IMT.

## Introduction

The intestinal microbiome has an important function in the defense against infectious diseases. This defensive system includes a consortium of phylogenetically diverse commensal microbes, including bacteria and other components. Colonization resistance is the term used to describe the way in which the microbiome operates both directly and indirectly to prevent colonization and invasive infection from pathogens, as well as to provide immune regulation^1^.

One such direct route by which members of the intestinal microbiome may contribute to colonization resistance is through the production of bacteriocins/antimicrobial peptides (AMPs) and other proteins by commensal bacteria that may kill pathobionts and other competitors through different mechanisms, including attack on bacterial cell walls.^2^ As an example of such an AMP, Type VI secretion system (T6SS) is a protein translocation complex secreted by members of the *Bacteroidetes* that has wide-spanning functions in killing and reducing the function and colonization ability of invading pathogens.^3^ An alternative direct route is the ability of commensal bacteria to act in competition with pathogens for resources and niches, e.g. indigenous *E. coli* competing with pathogenic *E. coli* 0157 for the amino acid proline (which it can exploit to promote growth);^4^ in addition, *E. coli* Nissle 1917 is able to compete with *Shigella* and limit its ability to cause invasive disease within the gut wall.^5^ As an additional route, gut microbial metabolites may also directly impact upon the life cycle of pathogenic bacteria, including bile acids, tryptophan-based metabolites, and short chain fatty acids (SCFAs); SCFAs are by-products of bacterial fermentation from nondigestible carbohydrates, and can induce production of AMPs^6^ and inhibit growth and fitness of pathogens, both directly^7^ and via routes including intracellular acidification.^8^

Indirect mechanisms of colonization resistance include microbiome-mediated regulation of the integrity of the gut barrier function to prevent penetration/ translocation of potential pathogens.^9^ Mucins are glycoproteins which act to protect the gut barrier against inflammation and colitis.^10^ Pathogens such as *Clostridioides difficile* are recognized to decrease the level of the major intestinal mucin, *muc2;*^11^ conversely, the commensal bacterial species, *Bifidobacterium longum*, restores growth of mucin.^12^

Modulation of innate and adaptive immune cells to enhance mucosal immunity is also an important role of microbially-secreted metabolites and microbial-associated molecular patterns (MAMPs).^13–15^ Toll-like receptors maintain intestinal homeostasis via their interaction with commensal bacteria.^16^ SCFAs including butyrate have a role in providing an energy source for intestinal epithelial cells as well as influencing T helper cell responses.^17^ In the presence of commensal bacteria, dendritic cells selectively induce immunoglobulin A (IgA), which also has an important immune function in prevention against invasive disease.^18^

Perturbation of the intestinal microbiota can be driven by factors such as medications (including antibiotics, opioids, immunosuppressive agents, and chemotherapeutics), diet, surgery, host immune status, and comorbid conditions.^19^ Such disruption of the microbiota not only alters its composition, but additionally may reduce the protective functions that it provides, including colonization resistance. Microbiome disruption and loss of colonization resistance is recognized to increase the risk of pathogens causing invasive disease and aberrant immune responses.

From a clinical viewpoint, manipulation of the gut microbiome to counter this perturbation and restore premorbid microbiome functionality is a relatively novel approach to reinstate colonization resistance, and may be a strategy that could be exploited for the treatment of particular infectious diseases; such new approaches are of particular pertinence and interest in an era of rising antimicrobial resistance. Gut microbiome manipulation strategies that have been explored encompass several modalities, such as prebiotics, probiotics, phage therapy, dietary manipulation, and fecal (or intestinal) microbiota transplantation (FMT).^20^ There are two particular attractions about FMT as an approach as a ‘microbiome therapeutic.’ Firstly, from a theoretical perspective, this is a ‘whole microbiome’ approach, attempting to rest and restore both the entire composition and functionality of an ecological community. Secondly, from a clinical perspective, there is already sound evidence from the scenario of recurrent *C. difficile* infection (rCDI) that this approach may be highly effective and overall safe. In this review, we will discuss the rationale and utility of FMT in a range of infectious diseases and potential further applications.

## Fecal (or intestinal) microbiota transplantation (FMT/IMT)

### Overview

Fecal microbiota transplantation (FMT; also known as ‘intestinal microbiota transplantation (IMT)’;^21^ see **Supplementary Material**) is the transfer of screened healthy donor stool to a recipient’s gastrointestinal tract via routes including nasogastric tube, enema, colonoscopy or capsules. The express aim of the procedure is manipulation of an affected intestinal microbiome to restore premorbid microbiome composition and function, as well aiding recovery of host-microbiome interactions.^22^ Importantly, good tolerability of the procedure has been seen in immunocompromised patients.^23^ FMT administration should strongly adhere to international guidelines to ensure donor blood and stool are screened for potentially transmissible pathogens.^24–26^ A fatality from an ESBL–producing *Escherichia coli* bacteremia transmitted from FMT donor stool in the United States has been previously reported after transmission to two patients that was not screened for ESBL producing organisms.^27^ Additionally, systemic infection from Shiga toxin–producing *Escherichia coli* (STEC) from a single donor to seven patients has also been reported.^28^ These recent complications have prompted the Food and Drug Administration to issue additional warnings regarding donor testing and quarantine.^29^ However, reassuringly, no significant delayed complications or adverse effects related to infections have been described in longitudinal studies looking at long term follow up of patients who have received FMT administration.^30–32^

**Figure 1.**
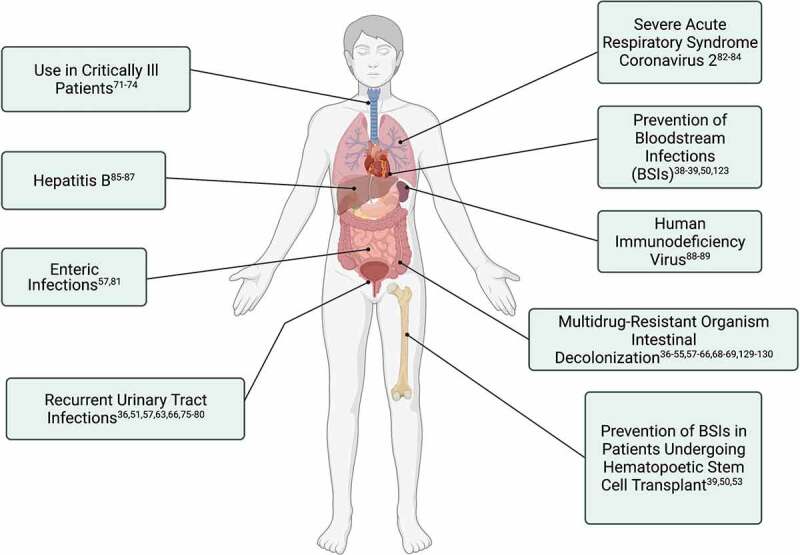
Uses of Fecal (or Intestinal) Microbiota Transplantation in the field of Infectious Diseases. Figure created with BioRender.com.

*C. difficile* infection (CDI) is a healthcare associated cause of diarrhea, precipitated by the use of antibiotics, and rCDI carries a significantly higher mortality than a single occurrence.^33^ FMT has been seen to confer a high success rate in the treatment of rCDI.^34^ The success in treatment of rCDI has led to a greater understanding of the wide interplay between the intestinal microbiome and defense against invading pathobionts and the role that FMT can play to restore and protect against invasive infection.^35^ The exploration into the mechanisms that contribute to the success of FMT in this field has led to the potential role of FMT to be explored in the treatment or prevention of other diseases with a link to the intestinal microbiome, including other infections. There currently exists an urgent need to seek non-antimicrobial options to address infectious diseases due to the global epidemic of antimicrobial resistance. Although life-saving, antimicrobials impact on the function of the intestinal microbiome and the subsequent long-term health consequences of their use is also increasingly being recognized, therefore FMT as a modality to restore the body’s own protection against invasive infection is of great interest. Figure 1 provides an overview of potential targets of FMT in management of infections and Table 1 summarizes the human intervention studies to date (animal studies related to the potential utility of fecal transplantation in infectious diseases have been recently comprehensively reviewed elsewhere in this journal,^90^ and are therefore not reviewed further here).

**Table 1.** Outcomes of studies utilizing fecal (or intestinal) microbiota transplantation in the treatment of human infectious diseases

Reference	Author	Year	No. of patients	Delivery method	Indication for FMT	Outcome post FMT	Follow up period
MDRO decolonization							
^36^	Aira	2020	1	colonoscopy	rCDI with recurrent UTIs (3 episodes in one year)	Reduction of intestinal *Enterobacteriaceae* from 74% to 0.07%	n/a
^37^	Baron	2019	1	nasogastric tube	CPE colonization with osteitis infection	Stool negative for CPE	12 months
^38^	Bar-Yoseph	2021	15	oral capsules	Intestinal colonization with MDRO	Stool negative for CPE 10/24 (41.7%) in control versus 9/15 (60%) in FMT group 8/12 (66.7%) negative in FMT group	1 month
^39^	Battipaglia	2019	10	enema or nasogastric tube	Allo-HSCT colonized with CPE/VRE or ESBL	Stool negative for MDRO in 7/10 (70%) patients	4–40 months
^40^	Biliński	2016	1	nasoduodenal tube	Multiple myeloma plus autologous HSCT colonized with CPE and ESBL-E	Stool culture negative for MDRO PCR positive for NDM	26 days
^41^	Biliński	2017	20	nasoduodenal tube	1) Hematological malignancy 2) Lung cancer 3) Renal transplant all colonized with CPE	Rectal swab negative for MDRO in 13/14 (93%)	6 months
^42^	Crum-Cianflone	2015	1	colonoscopy	Sacral wound plus spinal epidural abscess with rCDI colonized with multiple MDROs	Reduction from 24 MDROs pre-FMT to 11 post-FMT detected on culture	15 weeks
^43^	Davido	2017	8	nasogastric tube	Colonization with MDRO only	Rectal swab negative for CRE 3/8 (37.5%)	3 months
^44^	Davido	2019	8	nasoduodenal tube	Chronic renal failure colonized with VRE	Rectal swab negative for CRE 7/8 (87.5%)	3 months
^45^	Dias	2018	2	n/a	rCDI colonized with CPE	Rectal swab negative for CPE in 2/2 (100%) patients	3 months
^46^	Dinh	2018	17	nasogastric tube	Colonization with MDRO only	Rectal swab negative for CRE 4/8 (50%) Rectal swab negative for VRE 7/8 (87.5%)	3 months
^47^	Eysenbach	2016	15	n/a	rCDI colonized with VRE	Negative stool for VRE in 4/4 (100%) in IMT group versus 6/7 (86%) in control group	6 weeks
^48^	Freedman	2014	1	nasoduodenal tube	Hemophagocytic lymphohistiocytosis with CPE bacteremia and osteomyelitis	Stool cultures negative for CPE	8 months
^49^	García-Fernández	2016	1	colonoscopy	rCDI colonized with CPE	Stool cultures negative for CPE	6 months
^50^	Ghani	2021	17	nasogastric tube	1) Hematological malignancy colonized with MDROs 2) Renal transplant with recurrent MDRO UTIs 3) Recurrent MDRO UTI	Negative rectal swabs for MDRO in 7/17 (41.2%)	2 years
^51^	Grosen	2019	1	nasojejunal tube	Renal transplant with recurrent ESBL UTIs (7 hospital admissions in 5 months)	Stool negative for ESBL *Klebsiella pneumoniae*	8 months
^52^	Huttner	2019	22	nasogastric tube	1) Colonization with CPE 2) Invasive ESBL infection	Stool negative for MDROs in 9/22 (41%) in treatment group versus 5/17 (29%) in control	48 days
^53^	Innes	2017	1	nasogastric tube	Acute lymphoblastic leukemia undergoing allo-HSCT colonized with CPE	Stool negative for CPE	12 months
^54^	Jang	2015	1	nema and nasoduodenal tube	rCDI with spastic tetraplegia colonized with VRE	Stool positive for VRE	3 months
^55^	Jouhten	2016	n/a	colonoscopy	rCDI	Reduction in diversity of antibiotic resistant genes, except vanB	2 months
^56^	Lagier	2015	1	nasogastric tube	Nursing home resident colonized with CPE	Stool negative for CPE	14 days
^57^	Lahtinen	2017	4	colonoscopy	Recurrent ESBL *E. coli* UTIs	Stool cultures negative for ESBL-E	6 weeks
^58^	Leung	2018	8	enema	rCDI	Reduction in 95 antimicrobial resistance genes Increase in 37 resistance genes	90 days
^59^	Merli	2020	5	nasogastric tube	Pre-allo-HSCT 3/5 patients – carbapenem resistant Gram-negative bacteremia	Stool negative for CPE	113 days
^60^	Millan	2016	20	colonoscopy	rCDI	Reduced number and diversity of antibiotic resistant genes	1 year
^61^	Ponte	2017	1	nasoduodenal tube	rCDI colonized with CRE	3 stool samples negative for CRE	100 days
^62^	Saïdani	2019	10	nasogastric tube	rCDI colonized with CRE	Rectal swab negative for CPE/A in 8/10 (80%) FMT patients versus 2/10 (20%) in control group	14 days
^63^	Singh	2014	1	nasoduodenal tube	End-stage renal failure with recurrent ESBL *E. coli* transplant pyelonephritis	Perineal and throat swab positive for ESBL at 1 week, Negative at 2,4,12 months	12 months
^64^	Singh	2018	15	nasoduodenal tube	1) Renal transplant 2) Recurrent ESBL UTIs	Stool negative for ESBL in 3/15 (20%) after the first transplant 6/15 (40%) negative after the second transplant	4 weeks
^65^	Sohn	2016	3	enema	rCDI colonized with VRE	No eradication of VRE in 3/3	21 weeks
^66^	Stalenhoef	2017	1	nasoduodenal tube	Peritoneal dialysis with recurrent *Pseudomonas* UTIs	5 negative stool cultures for *Pseudomonas aeruginosa* Positive stool for ESBL *E. coli*	3 months
^67^	Stripling	2015	1	nasogastric tube	Renal and heart transplant with rCDI colonized with VRE	Decrease in abundance in stool of *Enterococcus* from 84% to 0.2% (7 weeks)	7 weeks
^68^	Su	2021	1	nasoduodenal tube	Acute myeloid leukemia post allo-HSCT colonized with CPE	Stool negative for CPE	26 months
^69^	Wei	2015	5	nasojejunal tube	MRSA enteritis post colorectal surgery	Stool negative for MRSA	3 months
Prevention of Clinical Infection in MDRO colonized patients							
^38^	Bar-Yoseph	2021	see *MDRO decolonization*			Death: 8/24 (33%) in control versus 0/15 in FMT group Clinical CPE infection: 9/24 (37.5%) in control versus 0/15 in FMT group	6 months
^39^	Battipaglia	2019	see *MDRO decolonization*			ESBL *E. coli* bacteremia in 1 patient No MDR bacteremia in 9/10 (90%) patients	90 days
^40^	Biliński	2016	see *MDRO decolonization*			No subsequent infections	26 days
^42^	Crum-Cianflone	2015	see *MDRO decolonization*			Reduction from five to one infective episodes	15 weeks
^48^	Freedman	2014	see *MDRO decolonization*			No subsequent infections	1.5 years
^50^	Ghani	2021	see *MDRO decolonization*			Significant reduction in inpatient bed days, bacteremia, and antibiotic use	2 years
^59^	Merli	2020	see *MDRO decolonization*			2/5 (40%) carbapenem resistant Gram-negative bacteremia	113 days
^67^	Stripling	2015	see *MDRO decolonization*			No further episodes of VRE sepsis	7 weeks
^68^	Su	2021	see *MDRO decolonization*			No CPE bacteremia	12 months
^70^	Gouveia	2021	1	colonoscopy and nasogastric tube	Recurrent ascending cholangitis (30 hospital admissions in 6 years) with recurrent MDR bacteremia	After 1st FMT: 3 hospitalisations with less resistant bacteria After 2 further FMTs: No further infections	4 months
Use in Critically Ill Patients							
^71^	Dai	2019	18	13: nasojejunal tube 4: gastroscopy 1: enema	Critically ill patients with antibiotic associated diarrhea	44.4% (8/18) resolution of abdominal symptoms and survival	12 weeks
^72^	Li	2015	1	nasoduodenal tube	Sepsis and severe diarrhea following vagotomy	Resolution of clinical symptoms	21 days
^73^	Li	2014	1	nasoduodenal tube	Sepsis and severe diarrhea in a patient with ulcerative colitis	Resolution of clinical symptoms	21 days
^74^	Wei	2016	2	nasogastric tube	Multiple organ dysfunction syndrome, septic shock, and severe watery diarrhea	Resolution of clinical symptoms in both	20 days
Recurrent Urinary Tract Infections (UTIs)							
^36^	Aira	2020	see *MDRO decolonization*			No further UTIs	12 months
^51^	Grosen	2019	see *MDRO decolonization*			One further ESBL UTI 6 days post FMT	12 months
^57^	Lahtinen	2017	see *MDRO decolonization*			1 episode of cystitis with fully sensitive organism	6 weeks
^63^	Singh	2014	see *MDRO decolonization*			No clinical infection	3 months
^66^	Stalenhoef	2017	see *MDRO decolonization*			No recurrent Pseudomonas infection One *E. coli* UTI	18 months
^75^	Biehl	2018	1	oral capsules	Renal transplant with recurrent ESBL *E. coli* UTIs (8 episodes over 2 years)	No further UTIs	9 months
^76^	Hocquart	2019	1	nasogastric tube	Irritable bowel syndrome with recurrent MDR *E. coli* UTIs (5 episodes in 6 months)	No further UTIs	8 months
^77^	Ramos-Martínez	2020	1	colonoscopy	rCDI with long-term suprapubic catheter and recurrent MDR *Pseudomonas* UTIs	No further UTIs	10 months
^78^	Steed	2020	10	nasogastric tube or colonoscopy	rCDI with MDR recurrent UTIs	Reduction in number of infections Improved resistance profile of positive isolates	one year
^79^	Tariq	2017	4	n/a	CDI with recurrent MDR UTIs (3–7 over a year)	0–4 UTIs No change in control group	one year
^80^	Wang	2018	1	colonoscopy	Recurrent ESBL *E. coli* UTIs (20 episodes over 23 months)	No further UTIs	25 months
Enteric infections							
^57^	Lahtinen	2017	3	colonoscopy	Chronic *Salmonella* carriage (×2) (A&B) Chronic variable immunodeficiency with chronic norovirus (C)	Patient A&B: 3× negative stool culture Patient C: no improvement in symptoms	up to 3 months
^81^	Soto	2019	2	oral capsules preceded by ertapenem	Immunocompromised patients with resistant *Salmonella infantis*	Resolution of clinical symptoms in both with negative stool culture	up to a year
Severe Acute Respiratory Syndrome Coronavirus 2							
^82^	Biliński	2021	2	nasojejunal tube	rCDI and COVID-19 infection	Full resolution from rCDI. Potential mitigation of adverse outcomes from COVID-19	30 days
^83^	Ianiro	2020	2	colonoscopy	rCDI and COVID-19 infection	Full resolution from COVID-19 and rCDI	8 weeks
^84^	Liu	2021	11	oral capsules	One-month post COVID-19 infection	Improvement in gastrointestinal symptoms and improvement in microbial diversity	One week
Hepatitis B							
^85^	Chauhan	2021	14	nasoduodenal tube (6 rounds every 4 weeks)	Chronic Hepatitis B (positive HBeAg)	HBeAg clearance in 2/12 (16.7%) versus 0/15 of comparator arm	6 months
^86^	Ren	2017	5	nasoduodenal tube (between 1–7 rounds every 4 weeks)	Chronic Hepatitis B (positive HBeAg)	Significant decline in HBeAg levels not seen in comparator arm	28–40 weeks
^87^	Xie	2018	5	nasojejunal tube (every 2 weeks)	Chronic Hepatitis B (negative HBeAg)	Significant decline in HBsAg levels not seen in comparator arm	n/a
Human Immunodeficiency Virus							
^88^	Serrano-Villar	2021	14	Weekly oral capsules for 8 weeks	HIV-infected on antiretroviral therapy	Sustained increase in bacterial diversity and reduction in intestinal fatty acid binding protein	48 weeks
^89^	Vujkovic-Cvijin	2017	6	colonoscopy	HIV-infected on antiretroviral therapy	Sustained increase in microbial diversity similar to donor stool. No change in inflammatory markers	24 weeks

Abbreviations: allo-HSCT – allogeneic hematopoietic stem-cell transplantation, COVID-19 – coronavirus disease 2019, CPE – carbapenemase-producing *Enterobacterales*, CRE – carbapenem-resistant *Enterobacterales*, ESBL – extended-spectrum beta-lactamase, ESBL-E – extended-spectrum beta-lactamase *Enterobacterales*, FMT – Fecal (or Intestinal) Microbiota Transplantation, HBeAg – hepatitis B virus e-antigen, HBsAg – hepatitis B surface antigen, HIV – human immunodeficiency virus, HSCT – hematopoietic stem-cell transplantation, MDR – multidrug-resistant, MDRO – multidrug-resistant organism, MRSA – methicillin-resistant *Staphylococcus aureus*, n/a – not available, NDM – New Delhi metallo-beta-lactamase 1, No. – number, rCDI – recurrent *Clostridioides difficile* infection, UTI – urinary tract infection, VRE – vancomycin-resistant Enterococci.

### Potential mechanisms of action of FMT/IMT

In the best-studied application of FMT to infectious disease – rCDI – it has been shown consistently that successful FMT is associated with the rapid and sustained restoration of a gut microbiome with high diversity and taxonomic profile similar to that of healthy donors.^91^ A relatively consistent finding between both CDI and non-CDI FMT studies is that high donor microbiota diversity and/or enrichment in particular commensal bacteria appear to be associated with FMT success.^92^ Studies in which either commensal bacteria cultured from healthy stool donors^93,94^ or spores derived from alcohol-shocked donor stool^95^ have been given as alternatives to conventional FMT in rCDI patients support the concept that transfer of commensal bacteria from donor to recipient is a central component of the efficacy of FMT, at least in this setting. However, the further demonstration in a pilot study that sterile, filtered FMT may have comparable efficacy to conventional FMT in the treatment of rCDI^96^ suggests that soluble components within FMT – including metabolites, microbial proteins, and/or bacteriophages and other nonbacterial microbiome components – may also be key mediators to the efficacy of FMT.

**Figure 2.**
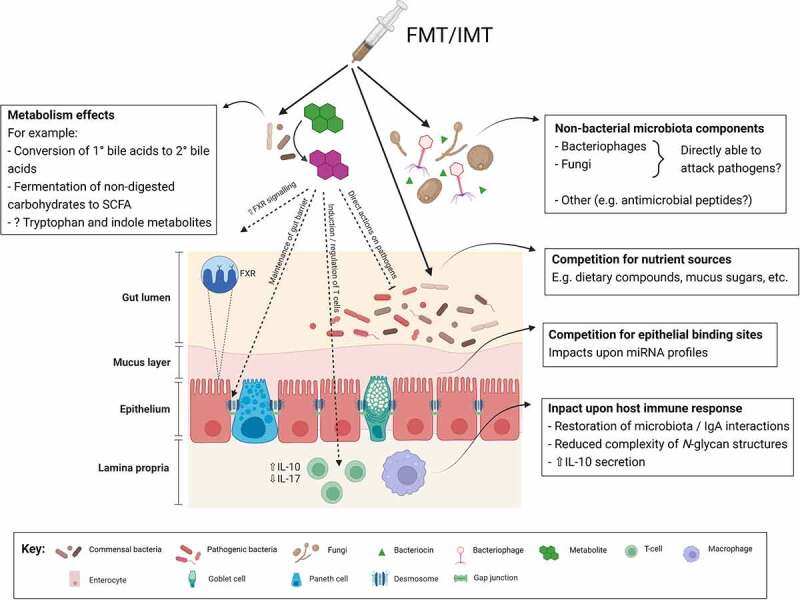
Proposed mechanisms of efficacy of fecal (or intestinal) microbiota transplantation in treating infectious diseases.Abbreviations: FMT: fecal microbiota transplant; FXR: farnesoid X receptor; IL: interleukin; IMT: intestinal microbiota transplant;miRNA: microRNA; SCFA: short chain fatty acids. Figure created with BioRender.com.

A summary of both established and proposed mechanisms of FMT is presented in Figure 2; many studies of such potential mechanisms have focused upon whether FMT may restore aspects of colonization resistance. For instance, the impact of FMT upon gut microbial metabolites has been extensively investigated.^97^ After FMT for rCDI, there is restoration of a range of SCFAs within the gut from very low levels up to levels similar to healthy donors;^98^ this includes the five carbon SCFA, valerate, which directly limits the growth of *C. difficile*.^7^ In addition, successful FMT is associated with the restoration of microbial bile salt hydrolases and an associated recovery of the premorbid gut bile acid milieu, removing bile acids which act as potential germination triggers (such as taurocholic acid) and restoring secondary bile acids which limit the growth of *C. difficile*.^99^ Such changes in gut microbial metabolites may act beneficially beyond just a direct effect upon a specific gut pathogen itself, and impact upon host responses more generally; for instance, FMT-related changes in gut bile acid profiles have also been associated with altered farnesoid X receptor-pathway signaling,^100^ and the secondary bile acids that are enriched post-FMT are associated with an impact upon regulatory T cells.^101^

A number of studies have described FMT-related changes in gut bacteriophage or fungal profiles, or defined profiles that predict treatment success, although these specific profiles are heterogeneous between conditions. For instance, while low relative abundance of Caudovirales bacteriophages in the gut predicts response to FMT in both patients with CDI and those with ulcerative colitis (UC),^102–105^ low gut levels of *Candida albicans* is associated with successful FMT in CDI patients^106^ but failure of FMT in UC.^107^ Given the established role of the virome in colonization resistance^108^ – including a role for bacteriophages in lysing infected cells and reducing bacterial fitness^109^ – there is a clear rationale as to why these changes in bacteriophage profiles may contribute to the efficacy of FMT.

FMT-related changes in host immune responses have also been increasingly well-defined; for instance, FMT for rCDI has been associated with restoration of gut bacteria-IgA interactions^110^ and may even reverse a CDI-related immunosenescent phenotype through its impact upon T cell repertoires.^111^ Both mouse and early human studies have associated successful FMT with increased interleukin-10 production by innate and adaptive immune cells, reduced interleukin-17 production, and reduced ability of macrophages, monocytes and dendritic cells to present MHCII-dependent bacterial antigens to colonic T cells.^112,113^ FMT-related changes in the gut microbiome have also been associated with changes in circulating and intestinal tissue microRNAs^114^ and reduction in complexity of serum *N-*glycan profiles toward that found in healthy donors,^115^ providing a potential link between the gut microbiome and epigenetic changes that may affect several aspects of host physiology, immune and otherwise.

There are grounds for expecting that FMT may also restore other aspects of colonization resistance, although there are limited data from human studies at present. For instance, FMT has been demonstrated to transfer bacteriocins in piglets,^116^ and commensal bacteria in FMT outcompete *C. difficile* for proline as an energy source in the gut of a mouse CDI model;^117^ however, comparable studies have not been published using human samples at present.

While stool derived from almost any donor who passes screening protocols appears to work effectively in FMT to treat recurrent CDI patients, experience of FMT in non-CDI conditions demonstrates much more heterogeneity in response overall.^92^ In addition to exploring microbiota ‘signatures’ of donors or recipients that may predict response to FMT, a further focus of research is investigation of microbiota functions that may also be predictive. Other potentially relevant factors relating to donors and recipients within this scenario may include genetics, immune status, and clinical factors (e.g. coexisting medications). Factors including modality used to prepare FMT, use of any gut preparation prior to the procedure and/or diet of donor and recipient may also be relevant to consider.^92^

## Potential clinical applications of IMT/FMT in infectious diseases:

### Multidrug-resistant organism (MDRO) decolonization

The intestinal microbiome is recognized to act as a reservoir for pathogens that carry antimicrobial resistant genes (ARGs), the collection of which is known as the resistome.^118^ Selection pressure from antimicrobials increases the genetic size of the resistome.^119^ Multidrug-resistant organisms (MDROs) are defined as bacteria resistant to more than three classes of antibiotics.^120^ Resistant ESKAPE pathogens that all colonise the intestine (***E****nterococcus faecium, **S**taphylococcus aureus, **K**lebsiella pneumoniae, **A**cinetobacter baumannii, **P**seudomonas aeruginosa*, and ***E****nterobacter* species) have been given priority status for new therapy development by the World Health Organization.^121^ The intestinal niche of MDROs has health implications to the host, as invasive infection can occur after translocation across the gut barrier or fecal contamination of other body sites. Infections from MDROs have poorer outcomes than infections sensitive to first line therapy, due to the poorer efficacy and worse toxicity of second line therapy, as well as the increased cost of these agents^122^ and this is of particular significance in immunocompromised patients where mortality with MDRO infections are significantly higher.^123^ Attempts to stop the spread of MDROs includes use of antimicrobial stewardship programs and hospital infection control procedures; however, more targeted therapies such as probiotics and selective digestive decontamination have had variable outcomes.^124^

Mouse models have provided proof of concept of the impact of FMT upon intestinal MDRO dynamics. For instance, administration to mice of FMT containing the commensal bacteria *Barnesiella* was associated with intestinal clearance of vancomycin-resistant enterococci (VRE);^125^ in addition, a four-strained consortium of commensal bacteria containing *Blautia producta* reduced susceptibility to VRE in a rodent model, with this protection attributable to production of a lantibiotic.^126^ Studies of FMT in the treatment of rCDI in humans have demonstrated a reduction in diversity and number of ARGs post-FMT;^55,58,60,127^ more recently, a similar finding has also been described in patients being treated with FMT for liver cirrhosis.^128^

Collectively, this evidence has led to the exploration of the use of FMT as a tool to “decolonize” the intestine to eradicate carriage of MDROs, which has been reported in a range of case reports, case series, and a single randomised trial (see Table 1).^36–46,48–52,54,56,61–66,68,69,129,130^ One of the perceived benefits of using FMT for this purpose has been that patients pose a lower nosocomial risk to others in a healthcare setting. Results from studies looking at intestinal decolonization of MDROs following FMT have been highly variable, in part due to the heterogeneity of the study design, patient cohorts, and FMT administration protocols.^131^ Biliński and colleagues reported decolonization rates or 75% in 20 patients,^41^ and Saïdani and colleagues also reported similarly high decolonization rates of 80% in 10 patients at 14 days (with a decolonization rate of 10% in a comparator arm). However, conversely, Davido and coworkers reported decolonization rate in eight patients as low as 37.5% after three months;^43^ in comparison, spontaneous decolonization rates for intestinal MDROs are reported as high as 48.2% after 90 days.^132^ The only reported randomized control trial (RCT) to date in this area demonstrated a non-significant decrease in rates of ESBL-E and CPE carriage in FMT-treated patients compared to the control group; in part, this was attributed to the low number of patients recruited and early termination of the trial by participants due to diarrhea.^52^ Although the reduction in the burden of ARG carriage in the gut has been described, the role of FMT as an infection control or an intestinal decolonization measure is still uncertain.

### Prevention of bloodstream infections (BSIs) in specific populations

In vulnerable populations with a disrupted gut microbiota (e.g. perturbed in terms of taxonomic profile or diversity of commensal bacteria), the risk of bloodstream infections (BSIs) has been noted to be increased.^133^ Studies reporting outcomes on the impact of FMT upon rCDI have reported a decrease in bloodstream infections (BSIs) post-FMT.^134^ Additionally, in studies investigating the impact of FMT in MDRO-colonized patients, a reduction in both MDRO-related and all-cause BSIs post-FMT has also been observed.^38,50^ The impact of FMT on the reduction of MDRO infections is currently being studied in two clinical trials, one looking specifically at patients with renal transplants (NCT02312986, NCT02922816).

In hematopoietic stem cell transplant (HSCT) patients – where preceding chemotherapy^135^ and the frequent need for broad-spectrum antibiotic therapy^136^ impacts the intestinal microbiome – lower intestinal microbial diversity is seen to correlate with worse survival post-HSCT.^137^ An associated increased risk of BSIs in HSCT patients with intestinal domination with Gram negative organisms is also seen, and increased mortality in those colonized with MDROs.^138–141^ In terms of infection outcomes, studies looking at the use of FMT in HSCT patients have noted a reduction in days of fever,^142^ and reduction in number of BSIs in HSCT post-FMT.^39,50^

Prevention of invasive disease in the Intensive Care Unit (ICU) setting using FMT has also been explored. Critical illness is recognized to dramatically impact the ecology of the microbial communities within the gut.^143^ The causes for disruption within the intensive care setting include hypoxic injury, enteral feeding, use of medications (such as proton pump inhibitors, antibiotics, and vasopressors), and intestinal dysmotility, collectively resulting in a reduction in diversity and beneficial functional output of commensal bacteria.^144^ These changes within the gut microbiome are associated with an increase in infectious complications and mortality in patients with severe systemic inflammatory response syndrome (SIRS).^145^ Mouse models have reported improved survival in septic mice following FMT with an improvement in the gut barrier function.^146,147^ To date, several case reports have noted a decrease in SIRS response including fever in patients in the intensive care setting following FMT;^72–74^ in addition, a case series of 18 ICU patients with antibiotic-associated diarrhea were treated with FMT, with full resolution of symptoms occurring in eight out of eighteen patients.^71^

Future targets to use FMT as a safe and cost-effective method to prevent BSIs could be aimed patient cohorts who are recognized to be at particular risk from their colonizing MDRO (utilizing a scoring system such as the INCREMENT score^148^), or cohorts recognized to be at increased risk of BSIs due to the risk of microbiome disruption related to preceding drug therapy (i.e. chemotherapy or prolonged antibiotics) or chronic disease.

### *Recurrent urinary tract infections (rUTIs*):

An important subgroup explored in the prevention of invasive infection is that of rUTIs. Non-antimicrobial options to treat rUTIs have limited evidence,^149^ and the risk of antimicrobial resistance increases with recurrent courses of anti-infectives.^150^ Increased abundance of uropathogenic organisms in the gut has been seen to be a direct risk factor for occurrence of UTIs with the same organism;^151^ therefore, re-establishment of the composition of the intestinal microbiome to restore colonization resistance and reduce the burden of invasive infection has been explored using FMT.

Patients who were treated with FMT for rCDI were also noted to have a reduction in their occurrence of rUTIs.^36,77,79,80^ Three case reports also describe use of FMT specifically to attempt to treat rUTIs, where no further UTIs were noted in patients after 8–12 weeks.^57,63,76^ FMT has also been used specifically to attempt to prevent rUTIs in renal transplant patients. These patients are recognized to have a lower intestinal microbial diversity than healthy controls^152^ and rUTIs are recognized to impact on the kidney graft function in these patients;^153^ as such, the restoration of the intestinal microbiota with FMT and prevention of invasive disease in this cohort could be of prognostic value. Two case reports and a case series have all reported a reduction in the occurrence of UTIs in renal transplant patients post-FMT despite no change in the risk factors predisposing the patients to recurrent infection.^50,51,75^

### Bacterial enteric infections

As seen in rCDI, the intestinal microbiota is the first line of defense against enteric infections, and a deeper understanding of the role of the gut microbiota has arisen from studying the relationship between these pathogens and commensal bacteria. Examples include the commensal *Blautia obeum*, which has been demonstrated to block infection from *Vibrio cholera* via hydrolysis of bile acids.^154^ Another commensal bacterium, *Clostridium scindens*, has been recognized to possess antimicrobial features against infection from *Entamoeba histolytica* and *C. difficile* via the biotransformation of primary to secondary bile acids.^155,156^ Mouse studies have shown that FMT reduced intestinal bacterial load of *Campylobacter jejuni*, a common cause of foodborne gastrointestinal infection, and additionally lowered cell damage caused by the bacteria, as well as susceptibility to the disease.^157,158^ In humans, successful eradication of chronic *Salmonella* infection after FMT has been reported twice in the literature, firstly in two patients with *Salmonella* infection alongside prolonged carbapenem usage^81^ and secondly as a modality to eradicate asymptomatic chronic *Salmonella* carriage in two patients where carriage had an impact on their occupation in the food industry.^57^ The exploitation of FMT to treat enteric infections in humans has not been fully explored to date; however, the restoration of microbiota-derived metabolites seen in preliminary studies of these infections have also been seen in the mechanisms explored as contributors to the success of FMT for rCDI.^35^ This modality therefore may have either a prophylactic role or use in chronic or relapsing infections where long term antibiotic use has a detrimental effect on the host.

### Viral infections:

The microbial communities in the intestine and respiratory system have a shared mucosal immune system that may be referred to as the ‘gut-lung axis,’ suggesting that there may be a role for FMT as a preventative or supportive measure in respiratory disorders.^159^ Infection with respiratory viruses such as respiratory syncytial virus and influenza have been noted in mouse models to result in transient changes in intestinal microbiota composition, with an increase in Bacteroidetes and a decrease in Firmicutes phyla abundance as well as increased levels of lipocalin-2 concentrations, suggesting inflammation, posing an increased potential risk of subsequent bacterial infection.^160^ A recent study indicated that patients with a higher load of severe acute respiratory syndrome coronavirus 2 (SARS-CoV-2) RNA in the stool had lower levels of commensal bacteria that produce protective metabolites such as short-chain fatty acids and tryptophan and higher levels of pathobionts such as *Collinsella aerofaciens* and *Morganella morganii*.^161^ A report on patients treated with FMT for rCDI during the coronavirus disease 2019 (COVID-19) pandemic noted full resolution from COVID-19 in two patients with concurrent CDI and COVID-19 infection.^83^ A further case series of two patients treated with FMT for rCDI with coexisting SARS-CoV-2 infection suggested that FMT may have had a role in the shortened recovery time seen in these patients.^82^ Another trial looked at administering FMT to patients one month following hospital discharge post-COVID-19, and noted improvement in both gastrointestinal symptoms and in microbial diversity.^84^

A similar ‘gut-liver axis’ is also purported to exist. Hepatitis B-infected patients with chronic carriage or decompensated liver cirrhosis are recognized to have a lower diversity of commensal bacteria and lower levels of microbial metabolites than healthy controls or those with asymptomatic hepatitis B carriage, which suggests that the gut microbiota may be responsible for modulating the effects of the virus on the liver.^162,163^ Response to treatment for hepatitis B is measured serologically using hepatitis B e-antigen (HBeAg) and hepatitis B surface antigen (HBsAg), where seroconversion of HBsAg is the ideal endpoint. Changes in HBeAg post-FMT has been seen in two case series: one with five patients (where decline in HBeAg was seen without any cases of seroconversion) and another study of twelve patients (where two patients had loss of presence of HBeAg, but no loss of HBsAg).^85,86^ A further case series reported HBsAg decline following FMT in HBeAg negative patients with a shift in the microbiota composition.^87^ Similarly, in human immunodeficiency virus (HIV) infection, disturbance of the microbiome is recognized to be related to virus response; the depletion of CD4+ cells is seen first in gut-associated lymphoid tissue, and this impacts on gut barrier function and Th17 cells function.^164–166^ A study of FMT administered to six macaques infected with simian immunodeficiency virus showed some immune restoration with significant increases in the number of peripheral Th17 and Th22 cells and reduced CD4 + T cell activation in gastrointestinal tissues, indicating some potential to enhance the immune system via T cell integrity. In humans, an early study looking at the use of FMT for rCDI in a severely immunocompromised patient noted an improvement in CD4+ counts as well as full recovery from rCDI.^167^ Sustained increase in microbial diversity similar to donor stool for up to 24 weeks was seen in another study of FMT administered to six HIV-infected patients – however, no changes were seen in inflammatory markers.^89^ A more recent study administered weekly oral capsules of FMT for eight weeks to 14 HIV positive patients and followed them up for 48 weeks; researchers described a sustained increase in bacterial diversity and reduction in intestinal fatty acid binding protein, which is recognized as a marker of gut barrier dysfunction.^88^ The clinical impact of this improvement in diversity is yet to be fully established in HIV patients. In terms of viral enteric infections, a case series of non-rCDI indications for FMT reported on a case attempting to trial FMT on a patient with chronic norovirus infection, without any change in their symptoms following FMT.^57^

## Conclusion:

As the understanding grows of the role of the intestinal microbiota in both the defense against infection but also as a potential reservoir of pathogens, so our knowledge also expands regarding postulated strategies for reversing a perturbed microbiome. The role for FMT in protecting against and treating infection appears to be most valuable in vulnerable cohorts and the scenario of chronic or relapsing infection; however, a limitation is that current evidence for a number of potential applications are at present derived from rodent studies only or small, early phase human studies. Although the administration of FMT is becoming more refined in terms of the availability and ease of capsulized administration, with further understanding of mechanisms of actions, refined bacterial consortiums that could potentially be personalized to target specific organisms would be of greater value, and would overcome the potential risks identified with FMT administration.^168^ Manipulation of the microbiota is an attractive target for infections due to the global effort to reduce the use of antibiotics and the worldwide antimicrobial resistance crisis; however, large scale RCTs are needed to confirm the true utility in each of these different conditions.

## Supplementary Material

Supplemental Material

## Data Availability

Data sharing is not applicable to this article as no new data were created or analyzed in this study.
