# Supplementary material for: The potential utility of fecal (or intestinal) microbiota transplantation in controlling infectious diseases
Source: Gut Microbes. 2022 Mar 1;14(1):2038856. doi: 10.1080/19490976.2022.2038856 (PMC8890388; doi:10.1080/19490976.2022.2038856)
Supplement: Supplemental Material [file KGMI_A_2038856_SM3991.docx]

**Supplementary Material:**

**The Potential Utility of Fecal (or Intestinal) Microbiota Transplantation in Controlling Infectious Diseases**

Rohma Ghani, Benjamin H. Mullish, Frances J. Davies, Julian R. Marchesi

1. **Nomenclature pertaining to Fecal or Intestinal Microbiota Transplantation.**

The terminology ‘Fecal Microbiota Transplantation (FMT)’ has been used in the main manuscript, as this is the one still of predominant use within published literature. However, there has been ongoing debate as to whether this should be the preferred terminology for the procedure, or an alternative term such as ‘Intestinal Microbiota Transplantation (IMT)’.

Primarily, it has been suggested that patient uptake and acceptability of the procedure may be improved by changing the name from “fecal” to “intestinal”^1^. It is also still biologically-accurate to describe the transplanted material as arising from the intestinal microbiota. The microbiota is the only component of the transplant recognized to engraft^2^, and as such it is not necessarily the feces that are being transplanted, but bacteria that are either mucosally-derived bacteria not attached to mucin, or sloughed off mucin to which mucosally-associated bacteria are attached. The origin of the bacteria may come from either the small or large intestine, which might be argued to give “intestinal microbiota” versus “fecal microbiota” more accuracy pertaining to the material transferred. The aim of FMT/IMT is to confer an intestinal bacterial community structure more similar to that of the healthy donor rather than the feces, viewed as a waste product of the human body^3^.

**References:**

1. Craven LJ, McIlroy JR, Mullish BH, Marchesi JR. Letter: intestinal microbiota transfer—updating the nomenclature to increase acceptability. Aliment. Pharmacol. Ther.2020; 52:1622–3.

2. Khoruts A, Brandt LJ. Fecal Microbiota Transplant: A Rose by Any Other Name. Off J Am Coll Gastroenterol | ACG 2019; 114.

3. Khoruts A, Bajaj JS. Reply to: “ ‘You know my name, but not my story’ – Deciding on an accurate nomenclature for faecal microbiota transplantation”: Intestinal microbiota transplantation: Naming a new paradigm. J Hepatol 2020; 72:1213–4.
